# Development of an Artificial Intelligence Model to Predict Endotracheal Intubation in Critically Ill Patients in Real Time

**DOI:** 10.3390/jcm15103642

**Published:** 2026-05-09

**Authors:** Da Hye Moon, Minkyu Kim, Seon-Sook Han, Tae-Hoon Kim, Dohyun Kim, Woo Jin Kim, Seung-Joon Lee, Yoon Kim, Jeongwon Heo, Hyun-Soo Choi, Yeonjeong Heo

**Affiliations:** 1 Department of Internal Medicine, Kangwon National University, Chuncheon 24341, Republic of Korea; ansekgo@naver.com (D.H.M.); ssunimd@kangwon.ac.kr (S.-S.H.); pulmo2@kangwon.ac.kr (W.J.K.); medfman@kangwon.ac.kr (S.-J.L.); 2 Department of Research & Development, Ziovision Co., Ltd., Chuncheon 24341, Republic of Korea; gim4855744@gmail.com (M.K.); dohyeon.kim@ziovision.co.kr (D.K.); yooni@ziovision.co.kr (Y.K.); 3 Institute of Medical Science, School of Medicine, Kangwon National University, Chuncheon 24341, Republic of Korea; blessing0104@naver.com; 4 Department of Computer Science and Engineering, Kangwon National University, Chuncheon 24341, Republic of Korea; 5 Department of Pulmonology, Chinjujeil Hospital, Jinju 52709, Republic of Korea; doctorhjw@naver.com; 6 Department of Computer Science and Engineering, Seoul National University of Science and Technology, Seoul 01811, Republic of Korea

**Keywords:** deep learning, mechanical ventilation, intensive care unit (ICU), artificial intelligence, endotracheal intubation, time-series prediction

## Abstract

**Background/Objectives**: In critically ill patients, endotracheal intubation (EI) is often performed to secure the airway or mechanical ventilation. Accurately predicting the timing of intubation significantly affects patient outcomes. We developed an artificial intelligence (AI) model designed for real-time risk stratification of patients requiring EI. **Methods**: We utilized the Medical Information Mart for Intensive Care-IV (MIMIC-IV) 2.2 dataset and performed model development using 15 clinical variables, including vital signs, Glasgow Coma Scale (GCS) score, and arterial blood gas analysis results. Patients intubated before or within 1 h of intensive care unit (ICU) admission were excluded. Clinical data from the ICU inherently consists of continuous time-series measurements. Traditional machine learning models often treat this information as static tabular data, neglecting vital temporal dynamics and patient history. Conversely, deep learning time-series approaches can capture these complex patterns over time. Thus, we applied the Gated Recurrent Unit with Decay++ (GRU-D++) model to predict the need for EI. GRU-D++ is an extension of the GRU and GRU-D. It builds upon the GRU-D to provide improved performance when handling datasets with exceptionally high rates of missing values. GRU-D++ is a time series deep learning model with an automatic mechanism for imputing missing values. This built-in capability eliminates the need for additional data preprocessing and has previously demonstrated high predictive performance. Using the 15 variables, we evaluated the optimal timing for EI in ICU-admitted patients by applying various AI models. **Results**: Among these, the GRU-D++ model demonstrated AUROC of 0.888, AUPR of 0.481, sensitivity of 0.474, specificity of 0.995, precision of 0.511, and F1 score of 0.491 on MIMIC-IV dataset. For KNUH dataset, the model demonstrated AUROC of 0.913, AUPR of 0.063, sensitivity of 0.162, specificity of 0.997, precision of 0.137, and F1 score of 0.147 within the 2 h in advance scenario. Furthermore, when compared with conventional scoring systems such as the Heart rate, Acidosis, Consciousness, Oxygenation, Respiratory rate (HACOR) score and Respiratory rate-Oxygenation (ROX) index, the GRU-D++ model also showed better performance predictive accuracy. **Conclusions**: The AI-based intubation prediction model developed in this study holds potential as a real-time risk stratification tool, providing timely risk assessments regarding the need EI. While operational threshold recalibration is essential prior to clinical deployment, further prospective multicenter studies are required to validate the clinical utility of this model in real-time practice.

## 1. Introduction

In patients admitted to the intensive care unit (ICU), endotracheal intubation is often performed to maintain the airway or perform mechanical ventilation. Endotracheal intubation and mechanical ventilation have a significant effect on the prognosis of patients admitted to the ICU. Various complications can occur as a result of tracheal intubation, and a high mortality rate has been reported. Among them, in critically ill patients, emergency tracheal intubation is associated with a significant frequency of complications [1].

In the ICU, scoring systems such as the Simplified Acute Physiology Score (SAPS) [2], and similar evaluation tools include Acute Physiology and Chronic Health Evaluation (APACHE) [3,4,5], Sequential Organ Failure Assessment (SOFA) [6,7] and Glasgow Coma Scale (GCS) [8,9] are widely utilized. Rather than serving as instruments for direct treatment decision-making, these tools are primarily designed for risk stratification and outcome prediction based on the patient’s physiological status and underlying conditions. However, they also have limitations, such as subjective interpretation due to the subjective measurement of some variables, which can undermine consistency in interpretation and cannot guarantee absolute prediction accuracy [10].

A notable limitation of these conventional indicators is that they are not specifically tailored to predict the optimal timing for endotracheal intubation, which we utilize in this study as a proxy for the initiation of mechanical ventilation (MV) in patients admitted to the ICU. To address this limitation, alternative tools such as the Heart rate, Acidosis, Consciousness, Oxygenation, Respiratory rate (HACOR) score [11] and the Respiratory rate-Oxygenation (ROX) index [12] have been introduced for application in patients who may require intubation. The HACOR score is designed to predict the likelihood of treatment success or failure in hypoxemic patients undergoing noninvasive ventilation. Similarly, the ROX index is used to assess the probability of treatment success or the need for intubation in patients with hypoxemic acute respiratory failure receiving heated humidified high-flow nasal cannula therapy. These indices provide clinical guidance in determining whether endotracheal intubation is necessary in hypoxemic patients.

The HACOR score is specifically validated for patients undergoing noninvasive ventilation, while the ROX index is optimized for those receiving high-flow nasal cannula therapy. While these indices provide valuable bedside guidance within their specific clinical contexts, they may not be directly applicable to the broader, undifferentiated ICU population who are not yet receiving these specific respiratory supports. In an effort to overcome these contextual limitations without overlooking the clinical utility of existing markers, we applied an artificial intelligence (AI) model to predict the need for endotracheal intubation in ICU patients. This approach aims to assist clinicians in making timely and accurate decisions regarding airway management.

Recent studies have shown that machine learning algorithms can effectively predict endotracheal intubation; however, there is still a need for models capable of making real-time predictions in a broader ICU patient population. In particular, research has been conducted to develop prognosis prediction models using large datasets that include patients’ vital signs and medical records [13,14]. These studies aim to predict the requirement for mechanical ventilation and offer practical support for clinical decision-making by physicians [15,16,17,18].

In recent years, machine learning techniques have been widely used to monitor patients’ conditions. The recently proposed Gated Recurrent Unit with Decay++ (GRU-D++) [19] model has demonstrated high predictive performance in the task of pressure ulcer prediction. In this study, we developed an early prediction system for mechanical ventilation using the time-series deep learning model GRU-D++. GRU-D++ was trained on the Medical Information Mart for Intensive Care-IV (MIMIC-IV) 2.2 dataset [20] and evaluated on both MIMIC-IV and Kangwon National University Hospital (KNUH) datasets. GRU-D++ can capture complex patterns in electronic health records and consider patient history, enabling our system to make more accurate predictions than existing models. Further, GRU-D++ automatically imputes missing values, reducing the need for physicians to address missing data manually. We provided a SHAP analysis to evaluate feature importance and demonstrate the effect of individual variables on the GRU-D++ predictions. Furthermore, we have verified that the variables identified as most influential by the SHAP analysis align closely with the expert opinions of our medical team.

This study aimed to utilize the previously established GRU-D++ AI model to identify optimal predictive variables for endotracheal intubation in patients admitted to the ICU. The ultimate goal is to facilitate timely and evidence-based clinical decision-making regarding the initiation of mechanical ventilation.

## 2. Materials and Methods

### 2.1. Study Design and Population

**Figure 1:**
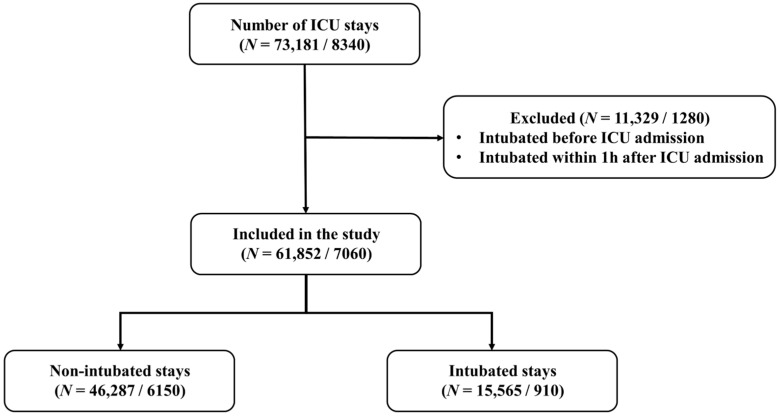
Flow diagram of cohort. “*N* = (MIMIC-IV/KNUH)” indicates the numbers of ICU stays in MIMIC-IV and KNUH datasets.

This was a retrospective observational cohort study. We retrospectively analyzed ICU patients from two datasets: the MIMIC-IV database (*n* = 61,852) and a Korean single-center cohort (KNUH, *n* = 7060), excluding those intubated within 1 h after ICU admission. The detailed process of patient selection and the final cohort construction for both datasets are illustrated in Figure 1. MIMIC-IV is a publicly available database collected from Beth Israel Deaconess Medical Center (Boston, MA, USA), containing 73,181 ICU stays. We excluded 109 stays that involved intubation before ICU admission. The KNUH dataset is in-house data collected from Kangwon National University Hospital (Chuncheon, Republic of Korea), the Republic of Korea, containing 8340 ICU stays. The KNUH dataset includes adult patients (age ≥ 18) admitted to the ICU from January 2016 to February 2023, excluding those intubated before ICU admission. The exclusion of patients intubated within 1 h of ICU admission was intended to focus the model on predicting subsequent clinical deterioration rather than identifying cases that required immediate emergency intervention upon arrival. By excluding these immediate cases, we aimed to evaluate the model’s ability to capture early warning signs in a more clinically stable population during the initial phase of their ICU stay.

#### Ethics Statement

This study was approved by the Institutional Review Board of Kangwon National University Hospital (Chuncheon, Republic of Korea) (KNUH-2023-10-004, 11 October 2023). All the procedures were conducted strictly in accordance with the approved protocol, adhering to standard regulation and the principles of Declaration of Helsinki. De-identified electronic medical record data from KNUH were used in the study, and as a result, the IRB of KNUH waived the requirement for informed consent.

### 2.2. Data Collection

In this study, we collected 15 variables, including demographics (age, sex), vital signs (body temperature, systolic BP, diastolic BP, heart rate, and respiratory rate), laboratory data (PaCO_2_, lactate, pH, and bicarbonate), GCS, including eye, verbal, and motor response components, and P/F ratio (ratio of partial pressure of oxygen [PaO_2_] to fraction of inspiratory O_2_ concentration [FiO_2_]). All variables were measured hourly and averaged if multiple measurements existed within 1 h.

The selection of these 15 variables was informed by their established clinical significance in predicting respiratory failure and the requirement for airway management, as evidenced by widely used clinical scoring systems such as the HACOR score (which includes heart rate, pH, GCS, P/F ratio, and respiratory rate) and the ROX index (SpO_2_/FiO_2_ to respiratory rate) [11,12]. Additionally, we prioritized variables that are routinely and reliably monitored in real-time ICU settings to ensure the model’s practical feasibility and to minimize the impact of missing data. Collectively, these variables represent the respiratory, hemodynamic, and neurological status, providing a comprehensive physiological snapshot for intubation decision-making.

Notably, although oxygen saturation (SpO_2_) was monitored and used as a foundational component to calculate the ROX index and P/F ratio, it was not included as a standalone input variable among the 15 selected features. This was a deliberate choice to ensure a more integrated and stable assessment of oxygenation status by accounting for FiO_2_ levels, thereby avoiding the potential confounding effects of oxygen therapy on raw SpO_2_ values.

### 2.3. Data Pre-Processing

The collected variables often contain missing values, which most machine learning models cannot handle directly. Therefore, we imputed missing values using the last observed value (i.e., forward filling), and any remaining missing values (no previously existing value) were imputed with the global mean of each variable (i.e., mean filling). Note that the GRU-D [21] and GRU-D++ models can automatically impute missing values using their internal mechanisms, so these imputations were not applied when using these models.

In the MIMIC-IV dataset, erroneous values exist within the blood gas variables. These erroneous values include partial pressure of arterial carbon dioxide (PaCO_2_) ≥ 0, PaCO_2_ ≤ 2583, pH ≥ 0, pH ≤ 999,999, bicarbonate ≥ 0, bicarbonate ≤ 999,999, PaO_2_ ≥ 0, and PaO_2_ ≤ 999,998. We treated these values as NULL (i.e., missing values). Within the raw MIMIC-IV database (prior to hourly aggregation), we identified 58 erroneous entries for PaCO_2_, 53 for pH, 27 for bicarbonate, and 59 for PaO_2_. Given the vast size of the dataset, these represent a negligible proportion of the total measurements, confirming that the overall data quality remains robust. Before inputting the variables into the machine learning models, we scaled the numerical variables to have a mean of zero and a variance of one. Also, we excluded cases where the ICU length of stay was shorter than the early prediction length used during model training.

### 2.4. Prediction Models

Electronic health records comprise a collection of continuously measured patient information. Thus, they can be viewed as a time series. However, most existing reports handle electronic health records as tabular data and neglect patients’ history information, such as previous vital signs and treatment history. In Addition, electronic health records have missing values that traditional machine learning models cannot directly utilize as input. Existing studies impute missing values using simple methods, such as forward fill or mean fill, which do not consider contextual information, such as patient history. To address this problem, we used the GRU-D++ model. GRU-D++ is a DL model for time series data that incorporates an automatic missing-value imputation mechanism inspired by the GRU-D model. However, GRU-D++ is more robust and effective when dealing with data exhibiting high rates of missing values.

The comparative methods used in this study were HACOR, ROX, logistic regression, decision tree, XGBoost, RNN, GRU, long short-term memory (LSTM), GRU-D, and GRU-D++. HACOR and ROX were specifically selected as clinical benchmarks because they are widely utilized, bedside-validated indices designed to guide decision-making for endotracheal intubation in patients with respiratory failure. Although these are traditional rule-based methods primarily developed for assessing the failure risk of noninvasive ventilation or high-flow nasal cannula therapy, comparing them with our AI model provides a direct clinical reference for the necessity of timely intubation. The ROX index is calculated using the following formula:

ROX index = (SpO_2_/FiO_2_)/Respiratory Rate

where SpO_2_ represents peripheral oxygen saturation (%), FiO_2_ is the fraction of inspired oxygen, and the respiratory rate is measured in breaths per minute.

Logistic regression, decision trees, and XGBoost are traditional ML models for tabular data. RNN, GRU, and LSTM are deep learning-based time series models. GRU-D and GRU-D++ are DL-based time series models that incorporate automatic missing-value imputation mechanisms. GRU-D incorporates temporal decay within its hidden states alongside the automatic imputation mechanism to handle missing values. Consequently, GRU-D demonstrates higher predictive accuracy than GRU when applied to datasets with missing values. However, the primary limitation of GRU-D is the requirement that variables in the initial time step be fully observed. GRU-D++ addresses this constraint by enhancing the imputation mechanism to accommodate missing values in instances where no prior observations exist. GRU-D++ is more effective and robust than GRU-D on datasets with high rates of missing values. For a fair comparison, we set all RNN models (RNN, GRU, LSTM, GRU-D, GRU-D++) to have similar representational power with a single layer of 32 hidden units and tanh activation. For XGBoost, we set num_boost_round to 1000, eta to 0.01, and max_depth to 6. All remaining hyperparameters were set to the default values provided by the xgboost package. All prediction models, including XGBoost and deep learning-based models (RNN, GRU, LSTM, GRU-D, and GRU-D++), were implemented using Python (version 3.9.12, Python Software Foundation, Wilmington, DE, USA).

During the preparation of this manuscript, the authors utilized ChatGPT (Plus, GPT-5.2) and Gemini (Pro, version 3.1) to improve the English language and readability. After using these tools, the authors reviewed and edited the content as needed and take full responsibility for the final version of the publication.

## 3. Results

### 3.1. Baseline Characteristics

The internal cohort, MIMIC-IV, consists of 61,852 patients (46,287 non-ventilated patients, 15,565 ventilated patients), whereas the external cohort, KNUH, consists of 7060 patients (6150 non-ventilated and 910 ventilated). In the internal cohort, we split the dataset into five folds. Four folds were used for training, and the remaining fold was used for test. Table 1 displays the baseline characteristics of both the internal and external cohorts.

For the binary variable, sex, we reported the number and proportion of male patients. For the numerical variables, we reported the median and interquartile range. To calculate the *p*-values, we used the chi-squared test for the binary variable and the Mann–Whitney U test for the numerical variables, since all numerical variables were found to deviate from normal distribution based on three normality tests (*p* < 0.05), Kolmogorov–Smirnov, Normal, and Jarque–Bera tests. Exact *p*-values can be found in Table A7.

### 3.2. Predictive Performances of Machine Learning Models

We developed 2, 4, and 8 h early prediction systems for determining the need for MV using various ML models and conducted experiments to compare their performances. Table 2 presents the predictive performances of these systems on the MIMIC-IV and KNUH datasets. We used the area under the receiver operating characteristic curve (AUROC) as an evaluation metric. The internal validation dataset (MIMIC-IV) was split into five folds; four folds were used for training, and one fold was used for testing, with this process repeated until all folds were tested. Additionally, 10% of the training set was used for model selection. The GRU-D++ model achieved an AUROC of 0.888 in internal validation and 0.913 in external validation on a 2 h early prediction scenario, making it the best performer in our experiments. Table A1, Table A2, Table A3, Table A4, Table A5 and Table A6 show the other evaluation metrics including AUPR, sensitivity, specificity, precision, and F1 score. The classification threshold for each model was calibrated to maximize the F1 score.

### 3.3. Comparison with HACOR Score and ROX Index

**Figure 2:**
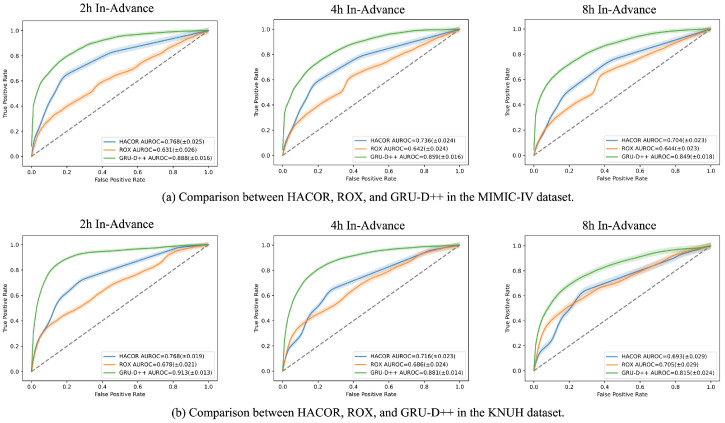
Comparison of AUROCs between GRU-D++ and conventional clinical indices, HACOR and ROX. The visualizations illustrate AUROC trends across both the MIMIC-IV and KNUH datasets for the 2-, 4-, and 8 h in-advance prediction scenarios. The higher positioning of the GRU-D++ curves compared to the HACOR and ROX over all scenarios indicates superior predictive capability of GRU-D++. AUROC, Area Under the Receiver Operating characteristic Curve; HACOR, Heart rate, Acidosis, Consciousness, Oxygenation, Respiratory rate; ROX, Respiratory rate-Oxygenation; GRU-D++, Gated Recurrent Unit with Decay++; MIMIC-IV, Medical Information Mart for Intensive Care-IV; KNUH, Kangwon National University Hospital. The dashed diagonal line indicates the performance of a random classifier (AUROC = 0.5).

In addition to comparing various machine learning models, we evaluated GRU-D++ against conventional clinical warning scores, specifically the HACOR score and the ROX index. The HACOR score predicts failure of noninvasive ventilation, whereas the ROX index predicts failure of high-flow nasal cannula therapy. Figure 2 displays the AUROCs of GRU-D++, the HACOR score, and the ROX index. The results indicate that GRU-D++ outperforms both the HACOR score and the ROX index across all early prediction scenarios. Also, Table 3 shows the *p*-values of DeLong test between the AUROCs of GRU-D++ and those of HACOR/ROX, confirming that the performance improvements achieved by GRU-D++ over HACOR/ROX are statistically significant.

### 3.4. Warning Score Analysis

**Figure 3:**
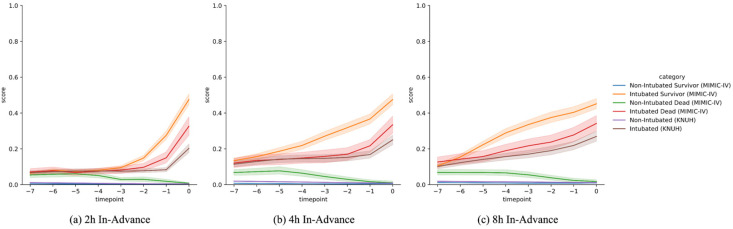
This figure plots the mean risk scores generated by the GRU-D++ over the 8 h preceding a clinical event (either endotracheal intubation or ICU discharge). For the MIMIC-IV dataset, trends are displayed for four subgroups: non-intubated survivors, non-intubated deaths, intubated survivors, and intubated deaths. For the KNUH dataset, patients are categorized into non-intubated and intubated groups. The results express how the predicted risk scores increase as the time to intubation nears for intubated patients and decrease as discharge approaches for non-intubated patients. GRU-D++, Gated Recurrent Unit with Decay++; MIMIC- IV, Medical Information Mart for Intensive Care-IV; KNUH, Kangwon National University Hospital.

Figure 3 shows the mean of predicted warning scores for GRU-D++ over the 8 h prior to discharge or intubation. To analyze patterns across different patient groups, we divided the MIMIC-IV data into four subgroups: non-intubated ICU survivors, non-intubated ICU deaths, intubated ICU survivors, and intubated ICU deaths. For the KNUH data, we divided the dataset into two groups, non-intubated and intubated, since ICU mortality information was unavailable. Our developed system successfully captured meaningful patterns from the data. For intubated patients, the warning scores increased as the time to intubation approached. Conversely, for non-intubated patients, the warning scores decreased as discharge time approached. Additionally, in the non-intubated group, the scores for deceased patients were higher than those for survivors, indicating that the system could potentially identify patients whose condition might improve with MV.

### 3.5. SHapley Value Analysis

**Figure 4:**
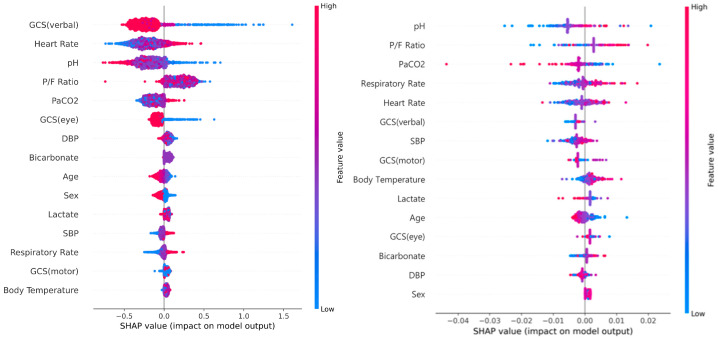
SHAP analysis illustrating the impact of each variable on GRU-D++ model predictions on MIMIC-IV (**left**) and KNUH (**right**). SHAP, Shapley Additive exPlanations; GCS, Glasgow Coma Scale; P/F Ratio, PaO_2_/FiO_2_ ratio; SBP, Systolic Blood Pressure; DBP, Diastolic Blood Pressure.

We obtained the importance of input variables from the trained GRU-D++ model using SHapley Additive exPlanations (SHAP) [22]. Figure 4 displays SHAP values of input variables. GCS (verbal) and heart rate were identified as the two most important factors influencing the application of MV. In addition, oxygen-related variables, such as pH, P/F ratio, and PaCO_2_, were also found to be significant factors. These findings align with established clinical knowledge.

## 4. Discussion

### 4.1. Main Findings

This study demonstrates that the GRU-D++ model provides superior predictive accuracy for endotracheal intubation in critically ill patients compared to both machine learning baselines and traditional scoring systems. The model achieved high AUROCs of 0.888 (MIMIC-IV) and 0.913 (KNUH) in the 2 h-advance prediction scenario. Unlike the HACOR score and ROX index, which showed limited performance in our diverse cohort, the GRU-D++ model maintained robust performance across both internal and external datasets. These findings suggest that our AI-based approach can effectively support clinicians in making timely and objective decisions for airway management.

### 4.2. Comparison of Predictive Performance and Clinical Utility

In this study, we applied the GRU-D++ model—an advanced deep learning algorithm designed to handle missing values in clinical datasets—for the prediction of endotracheal intubation in ICU patients. By utilizing 15 clinical variables, including vital signs, GCS, and arterial blood gas analysis results, we aimed to identify the optimal timing for endotracheal intubation. Among the various AI models tested, the GRU-D++ consistently showed better predictive performance across both datasets. The decision tree demonstrated poor predictive performances. The poor performance of a single decision tree on unseen test data is a well-documented phenomenon; this model tends to learn rigid, step-like functions that overfit the training dataset. Consequently, it fails to generalize to test data when the underlying clinical dataset is highly complex or exhibits distributional shifts. Notably, its performance also surpassed that of conventional clinical scoring systems such as the HACOR score and ROX index.

However, the comparison between our model and these clinical scores should be interpreted with caution. The HACOR and ROX scores were originally developed and validated for specific clinical contexts—patients receiving non-invasive ventilation (NIV) and high-flow nasal cannula (HFNC), respectively. In contrast, our study included a more heterogeneous population of critically ill patients regardless of their initial respiratory support method. Despite these differences in their developmental backgrounds, the superior performance of our GRU-D++ model suggests its potential as a more versatile and robust tool in general ICU settings. While the numerical differences in AUROC might appear incremental, the clinical significance lies in providing continuous, automated monitoring that reduces the subjective variability inherent in manual scoring systems [23,24]. Importantly, our model should be interpreted as a probabilistic decision-support tool predicting the likelihood of a clinical decision, rather than a measure of absolute physiological necessity.

Specifically, in the 2 h advance prediction scenario, the GRU-D++ model achieved high AUROCs of 0.888 for the MIMIC-IV dataset and 0.913 for the KNUH dataset. These results significantly outperformed the conventional HACOR score (0.768 for MIMIC-IV; 0.768 for KNUH) and ROX index (0.631 for MIMIC-IV; 0.678 for KNUH) across both cohorts.

### 4.3. Mechanistic Explanation and Advantages of GRU-D++ in Real-World Scenarios

We conducted experiments to evaluate the performance of various methods, including traditional warning scores and machine learning models for the early prediction of mechanical ventilation. GRU-D++ demonstrated improved performance over all competing methods on both internal and external datasets. This improvement stems from the internal missing-value imputation mechanism within GRU-D++. Conventional methods require missing values to be imputed prior to model input, whereas GRU-D++ automatically imputes missing values with optimized estimations. From a physiological perspective, this mechanism incorporates a ‘decay effect’ that accounts for the time elapsed since the last observation, allowing the model to capture the dynamic ‘trajectory’ of respiratory failure more accurately than static scoring systems. This advantage enhances predictive performance and reduces preprocessing effort. Hence, we anticipate that our system is advantageous in real-world scenarios.

### 4.4. Clinical Significance of Granular Time-Point Prediction

Previous studies have also demonstrated the feasibility of predicting the need for endotracheal intubation by applying AI models to data extracted from the MIMIC-III database [15,25]. These studies highlight the potential of AI-based models to support real-time decision-making regarding endotracheal intubation during the early period of ICU admission, by showing prediction performance with AUC of 0.86 and 0.97 respectively.

However, a key strength of our study, relative to previous works, lies in its ability to estimate probabilities of intubation necessity at more granular time points—specifically at 2, 4, and 8 h after ICU admission. By using the GRU-D++ model developed in our study, clinicians can anticipate the likelihood of intubation as early as 2 h after ICU admission. This enables proactive preparation for airway management, such as preparing for video laryngoscopy in case of a difficult airway, or alerting backup personnel in advance, thereby improving the safety and efficiency of clinical interventions. To operationalize this in clinical practice, the system can be configured to trigger real-time alerts for the attending physician or the rapid response team when the predicted probability exceeds a specific threshold, facilitating immediate bedside reassessment and improving the safety and efficiency of clinical interventions. While high discriminatory performance (e.g., AUROC) is a prerequisite for a predictive model, translating these metrics into tangible clinical utility is paramount. In practice, the GRU-D++ model is designed to function as an automated, continuous bedside monitoring tool. By continuously ingesting ongoing hourly clinical measurements, the system dynamically recalculates the patient’s risk of requiring endotracheal intubation. From a workflow perspective, the model can be calibrated to a specific operational threshold (e.g., the threshold maximizing the F1 score) to trigger real-time, automated alerts for the attending physician or rapid response team. This early warning system facilitates immediate bedside reassessment, proactive resource allocation, and advanced preparation for airway management.

Although this model demonstrates predictive potential, its clinical application could involve a system that provides real-time information to the attending physician or rapid response team. Future studies are required to determine optimal clinical thresholds and to validate the efficacy of such real-time alert systems in actual bedside settings.

### 4.5. Generalizability Across Diverse ICU Populations

To date, among the available clinical scoring systems, only the HACOR score and ROX index have been commonly used to assist in predicting the need for intubation. However, our AI-based model offers enhanced temporal resolution and potential for real-time clinical application, representing a significant advancement in the early detection of respiratory failure in critically ill patients.

These two indices have been used to assist clinical decision-making regarding whether to proceed with endotracheal intubation in patients with hypoxemia [23,24]. However, the HACOR score is limited to use in hypoxemic patients only, and the ROX index is applicable specifically to those receiving high-flow nasal cannula therapy, making it unsuitable for broader ICU populations. To overcome these limitations, we aimed to develop an AI-based model capable of predicting the need for intubation in all patients admitted to the ICU, regardless of oxygen delivery method or the presence of hypoxemia. Our model was trained using data that included not only hypoxemic patients but also the broader ICU population. Also, it incorporated various oxygen delivery methods prior to intubation, including nasal prongs, simple oxygen masks, and high-flow nasal cannula, thereby increasing its generalizability and clinical applicability for real-time decision support in diverse ICU settings. Consistent with recent clinical AI trends [26,27], the model moves beyond purely numerical performance to provide actionable decision support, bridging the gap between predictive algorithms and practical ICU management.

Interestingly, the predictive performance in the external validation cohort (KNUH AUROC 0.913) exceeded that of the internal cohort (MIMIC-IV AUROC 0.888). This difference likely stems from the inherent nature of the datasets. While MIMIC-IV is a highly heterogeneous, multi-departmental database collected over a decade, the KNUH cohort reflects the more consistent clinical protocols and intubation thresholds of a single institution. This relative homogeneity in the external dataset likely allowed for clearer identification of predictive patterns. Furthermore, geographical differences in ICU practices—such as varying thresholds for respiratory intervention between the US and South Korea—may have contributed to the higher predictability in the KNUH dataset. These findings underscore the model’s robust generalizability and its potential for high performance when adapted to specific clinical environments.

However, we acknowledge the relatively low sensitivity (0.162) and AUPR observed in the KNUH cohort at the F1-optimal threshold. This suggests that while the model effectively minimizes false alarms, it may miss critical intubation events if deployed with a rigid threshold. Therefore, the model is more accurately described as a risk-stratification tool, and operational threshold recalibration is essential prior to clinical deployment to optimize sensitivity based on institutional needs.

### 4.6. Interpretation of Predicted Warning Scores

In our study, we analyzed the average predicted warning scores, as visualized in Figure 2, generated by the GRU-D++ model during the 8 h preceding either discharge or EI. Among the intubated group in the MIMIC-IV dataset, survivors showed a gradual increase in warning scores as the time of intubation approached, supporting the validity of our predictive model. In addition, in the non-intubated group, deceased patients exhibited higher risk scores than those who survived, suggesting that the model successfully identified clinical deterioration in these high-risk patients, even in the absence of a subsequent intubation event.

## 5. Limitations

This study has several limitations. First, the missing rates for certain arterial blood gas variables, such as PaCO_2_ (14.38%) and pH (13.28%), were relatively high. This is primarily because we only included data collected after ICU admission and prior to endotracheal intubation. Notably, when data obtained prior to ICU admission were included, the missing rate for PaCO_2_ dropped significantly to 1.87%. The higher missing rate in our dataset may reflect cases in which critically ill patients underwent arterial blood gas analysis immediately before ICU admission, leading to an early decision for intubation upon ICU entry, thereby limiting the availability of subsequent arterial blood gas measurements.

Second, we excluded patients who underwent intubation within 1 h of ICU admission. This decision was made because patients transferred to the ICU are often already in critical condition while in the emergency department or general ward, and many require immediate airway intervention upon arrival. Including these patients would limit the model’s ability to identify predictive markers before clinical deterioration, and thus, they were excluded to ensure more meaningful analysis of early predictors.

Third, the primary outcome—endotracheal intubation—reflects a clinical decision made by medical practitioners rather than a purely objective physiological event. Consequently, the model may partly capture physician behavior and localized practice patterns. However, we sought to address this by performing external validation with the KNUH dataset. The consistent predictive performance across two distinct cohorts from different geographical and clinical environments suggests that our GRU-D++ model identifies fundamental physiological trajectories leading to respiratory failure, rather than merely reflecting institutional biases.

Fourth, our model primarily relies on physiological variables and does not incorporate treatment-related factors, such as specific oxygen therapy modalities (e.g., HFNC, NIV) or pharmacological interventions. While incorporating these factors could provide additional clinical context, they are often recorded inconsistently across different electronic health record systems. We focused on core physiological trajectories, which inherently reflect the patient’s response to ongoing treatments. Nonetheless, future studies integrating real-time treatment data could further refine the model’s predictive accuracy and clinical relevance.

Fifth, the datasets employed in this study exhibit inherent class imbalance. Specifically, the intubated group represents approximately 25.0% of the MIMIC-IV cohort and 12.9% of the KNUH cohort. To assess model performance under conditions reflecting real-world ICU prevalence, the models were trained using raw clinical data distributions without the application of additional sampling or weighting techniques. Applying imbalance-mitigation strategies, such as SMOTE or cost-sensitive learning, is reserved for future investigation. This study is retrospective, and we only validated our results on a single external cohort, KNUH. Therefore, it is limited to generalize our findings to other cohorts. Prospective, multicenter clinical trials are required to fully validate the model’s generalizability and real-world efficacy across diverse ICU populations.

Sixth, our current evaluation focuses primarily on discriminatory metrics (e.g., AUROC, AUPRC). We did not perform a formal calibration assessment (such as calculating Brier scores or plotting calibration curves) or a Decision Curve Analysis (DCA). Evaluating the model’s calibration and assessing its net clinical benefit via DCA will be essential prior to clinical deployment. Additionally, our study predicts the specific clinical event of endotracheal intubation but does not incorporate longitudinal clinical outcome data, such as ventilator-free days, ICU length of stay, or long-term survival rates.

## 6. Conclusions

The AI-based intubation prediction model developed in this study holds potential as a real-time risk stratification tool, providing timely risk assessments regarding the need for endotracheal intubation. This model demonstrated superior performance compared to conventional indices such as HACOR and ROX scores. A specific clinical strength of this model is its ability to provide accurate predictions within a 2 h window, offering clinicians critical lead time. These findings suggest that the integration of AI into ICU workflows could optimize critical care management by providing objective, data-driven insights. While operational threshold recalibration is essential prior to clinical deployment, further prospective multicenter studies are required to validate the clinical utility of this model in real-time practice.

## Figures and Tables

**Table 1:** Baseline characteristics of MIMIC-IV and KNUH datasets.

	MIMIC-IV				KNUH			
	All (*N* = 61,852)	Non Intubated (*N* = 46,287)	Intubated (*N* = 15,565)	*p*-Value	All (*N* = 7060)	Non Intubated (*N* = 6150)	Intubated (*N* = 910)	*p*-Value
Age	67.58 (55.56–78.91)	67.58 (55.53–78.94)	67.38 (57.31–76.71)	<0.05	73.00 (57.00–81.00)	73.00 (57.00–81.00)	74.00 (60.00–81.00)	<0.05
Sex	1,308,153 (53.79%)	1,288,589 (53.67%)	19,564 (62.85%)	<0.05	303,429 (53.42%)	302,311 (53.39%)	1118 (61.43%)	<0.05
Body Temperature	36.78 (36.56–37.06)	36.78 (36.56–37.06)	36.61 (36.20–37.06)	<0.05	36.80 (36.43–37.20)	36.80 (36.47–37.20)	36.50 (36.00–37.20)	<0.05
SBP	118.00 (104.00–134.00)	118.00 (104.00–134.00)	115.00 (102.00–131.00)	<0.05	126.00 (112.00–141.24)	126.00 (112.00–141.00)	127.00 (103.50–150.00)	0.96
DBP	64.00 (54.00–74.00)	64.00 (54.00–74.00)	61.00 (52.50–72.00)	<0.05	70.00 (61.00–80.00)	70.00 (61.00–80.00)	72.00 (60.00–85.81)	<0.05
Heart Rate	83.00 (71.00–96.00)	83.00 (71.00–96.00)	87.00 (77.00–103.00)	<0.05	85.00 (73.00–98.00)	85.00 (73.00–98.00)	104.00 (87.00–118.58)	<0.05
Respiratory Rate	19.00 (16.00–22.00)	19.00 (16.00–22.00)	17.00 (15.00–22.00)	<0.05	20.00 (16.00–23.00)	20.00 (16.00–23.00)	22.50 (18.00–28.00)	<0.05
PaCO_2_	41.00 (35.00–48.00)	41.00 (35.00–48.00)	42.50 (38.00–48.00)	<0.05	30.30 (26.20–36.70)	30.30 (26.20–36.60)	34.40 (26.60–41.50)	<0.05
Lactate	1.80 (1.20–2.60)	1.70 (1.20–2.50)	2.40 (1.80–3.40)	<0.05	1.20 (0.80–1.90)	1.20 (0.80–1.90)	2.30 (1.20–4.80)	<0.05
pH	7.38 (7.32–7.42)	7.38 (7.33–7.43)	7.35 (7.29–7.40)	<0.05	7.43 (7.38–7.47)	7.43 (7.38–7.47)	7.36 (7.26–7.44)	<0.05
Bicarbonate	24.00 (21.00–27.00)	24.00 (21.00–27.00)	22.00 (20.00–25.00)	<0.05	20.90 (17.70–24.20)	20.90 (17.80–24.20)	19.60 (15.15–23.30)	<0.05
P/F Ratio	328.57 (263.89–346.43)	328.57 (266.67–346.43)	232.50 (117.50–306.25)	<0.05	339.29 (247.50–395.83)	339.29 (252.50–395.83)	153.33 (98.83–244.17)	<0.05
GCS (motor)	6.00 (6.00–6.00)	6.00 (6.00–6.00)	4.00 (1.00–6.00)	<0.05	6.00 (5.00–6.00)	6.00 (5.00–6.00)	2.00 (1.00–5.00)	<0.05
GCS (verbal)	5.00 (4.00–5.00)	5.00 (5.00–5.00)	0.00 (0.00–4.00)	<0.05	4.00 (3.00–5.00)	4.00 (3.00–5.00)	3.00 (1.00–4.00)	<0.05
GCS (eye)	4.00 (4.00–4.00)	4.00 (4.00–4.00)	1.00 (1.00–4.00)	<0.05	4.00 (3.00–4.00)	4.00 (3.00–4.00)	1.00 (1.00–3.00)	<0.05

Abbreviations: MIMIC-IV, Medical Information Mart for Intensive Care-IV; KNUH, Kangwon National University Hospital; SBP, Systolic Blood Pressure; DBP, Diastolic Blood Pressure; P/F Ratio, PaO_2_/FiO_2_ ratio; GCS, Glasgow Coma Scale.

**Table 2:** AUROCs of various machine learning models and clinical scoring methods across different scenarios. For each dataset, results are shown for 2, 4, and 8 h early predictions. The results are expressed as the mean AUROC followed by the standard deviation (±SD), derived from five-fold cross-validation.

AUROC	MIMIC-IV			KNUH		
	2 h In-Advance	4 h In-Advance	8 h In-Advance	2 h In-Advance	4 h In-Advance	8 h In-Advance
HACOR	0.768 (±0.025)	0.736 (±0.024)	0.704 (±0.023)	0.768 (±0.019)	0.716 (±0.023)	0.693 (±0.029)
ROX	0.631 (±0.026)	0.642 (±0.024)	0.644 (±0.023)	0.678 (±0.021)	0.686 (±0.024)	0.705 (±0.029)
Logistic	0.736 (±0.030)	0.711 (±0.027)	0.689 (±0.023)	0.745 (±0.022)	0.706 (±0.026)	0.690 (±0.033)
Decision Tree	0.593 (±0.015)	0.593 (+-0.014)	0.569 (±0.012)	0.579 (±0.014)	0.564 (±0.013)	0.547 (±0.011)
Random Forest	0.820 (±0.023)	0.807 (±0.021)	0.764 (±0.021)	0.762 (±0.021)	0.755 (±0.021)	0.753 (±0.024)
XGBoost	0.867 (±0.017)	0.834 (±0.018)	0.802 (±0.021)	0.797 (±0.018)	0.756 (±0.021)	0.753 (±0.026)
RNN	0.762 (±0.022)	0.729 (±0.023)	0.784 (±0.022)	0.870 (±0.016)	0.807 (±0.020)	0.780 (±0.026)
GRU	0.805 (±0.017)	0.791 (±0.019)	0.812 (±0.019)	0.897 (±0.013)	0.854 (±0.017)	0.798 (±0.026)
LSTM	0.815 (±0.019)	0.793 (±0.020)	0.808 (±0.020)	0.893 (±0.013)	0.862 (±0.016)	0.805 (±0.024)
GRU-D	0.885 (±0.017)	0.857 (±0.017)	0.833 (±0.019)	0.856 (±0.018)	0.863 (±0.017)	0.774 (±0.028)
GRU-D++	0.888 (±0.016)	0.859 (±0.016)	0.849 (±0.018)	0.913 (±0.013)	0.881 (±0.014)	0.815 (±0.024)

Abbreviations: MIMIC-IV, Medical Information Mart for Intensive Care-IV; KNUH, Kangwon National University Hospital; HACOR, Heart rate, Acidosis, Consciousness, Oxygenation, Respiratory rate; ROX, Respiratory rate-Oxygenation; XGBoost, eXtreme Gradient Boosting; RNN, Recurrent Neural Network; GRU, Gated Recurrent Unit; LSTM, Long Short-Term Memory; GRU-D, Gated Recurrent Unit with Decay; GRU-D++, Gated Recurrent Unit with Decay++.

**Table 3:** Significance analysis (DeLong test) of AUROC improvements. Across all prediction scenarios, the resulting *p*-values were approximated 0.00, robustly confirming that the predictive performance of the GRU-D++ model is significantly superior to both the HACOR score and the ROX index.

		DeLong Test *p*-Value
2 h In-Advance	HACOR	0.00
	ROX	0.00
4 h In-Advance	HACOR	0.00
	ROX	0.00
8 h In-Advance	HACOR	0.00
	ROX	0.00

Abbreviations: HACOR, Heart rate, Acidosis, Consciousness, Oxygenation, Respiratory rate; ROX, Respiratory rate-Oxygenation.

## Data Availability

The data that support the findings of this study are available on request from the corresponding author. The data are not publicly available due to privacy or ethical restrictions.

## Abbreviations

The following abbreviations are used in this manuscript:

AUROC	Area under the receiver operating characteristic curve
DBP	Diastolic blood pressure
GCS	Glasgow coma scale
GRU	Gated recurrent unit
GRU-D	Gated recurrent unit with decay
GRU-D++	Gated recurrent unit with decay++
HACOR	Heart rate, acidosis, consciousness, oxygenation, respiratory rate
KNUH	Kangwon National University Hospital
LSTM	Long short-term memory
MIMIC-IV	Medical Information Mart for Intensive Care-IV
P/F ratio	PaO_2_/FiO_2_ ratio
RNN	Recurrent neural network
ROX	Respiratory rate–oxygenation
SBP	Systolic blood pressure
SHAP	Shapley additive explanations
XGBoost	Extreme gradient boosting

## Appendix A

### Appendix A.1. Hyperparameters

(a) Logistic Regression

- penalty: l2
- C: 1.0
- max_iter: 1000

(b) Decision Tree

- criterion: gini
- splitter: best
- max_depth: None
- min_samples_split: 2
- min_samples_leaf: 1
- min_weight_fraction_leaf: 0.0
- max_features: None
- max_leaf_nodes: None
- min_impurity_decrease: 0.0

(c) Random Forest

- criterion: gini
- max_depth: None
- min_samples_split: 2
- min_samples_leaf: 1
- min_weight_fraction_leaf: 0.0
- max_features: sqrt
- max_leaf_nodes: None
- min_impurity_decrease: 0.0
- bootstrap: True
- oob_score: False
- max_samples: None

(d) XGBoost

- num_boost_round(n_estimators): 1000
- max_depth: 6
- max_leaves: 0
- max_bin: 256
- grow_polish: depthwise
- eta(learning_rate): 0.01
- objective: binary:logistic
- booster: gbtree
- tree_method: hist
- gamma(min_split_loss): 0
- min_child_weight: 1
- max_delta_step: 0
- subsample: 1
- sampling_method: uniform
- colsample_bytree: 1
- colsample_bylevel: 1
- colsample_bynode: 1
- lambda(reg_lambda): 1
- alpha(reg_alpha): 0
- scale_pos_weight: 1
- early_stopping_rounds: 20
- eval_metric: logloss
- num_parallel_tree: 1

(e) RNN, GRU, LSTM, GRU-D, GRU-D++

- learning rate: 0.001
- early stopping patience: 20
- Number of layers: 1
- Number of hidden units: 32
- Activation: tanh

### Appendix A.2. Additional Evaluation

**Table A1:** Comparison of various prediction methods on the MIMIC-IV dataset: 2 h in-advance scenario. Thresholds for the models are 0.070, 0.506, 0.336, 0.298, 0.510, 0.506, 0.516, 0.496, 0.510, 0.063, 10.410 (top to bottom).

	AUROC	AUPR	Sensitivity	Specificity	Precision	F1
LogisticRegression	0.736 (±0.030)	0.068 (±0.007)	0.145 (±0.005)	0.990 (±0.001)	0.154 (±0.017)	0.149 (±0.009)
DecisionTree	0.593 (±0.015)	0.099 (±0.005)	0.378 (±0.013)	0.985 (±0.000)	0.235 (±0.007)	0.290 (±0.008)
RandomForest	0.820 (±0.023)	0.461 (±0.009)	0.426 (±0.015)	0.996 (±0.000)	0.542 (±0.012)	0.477 (±0.006)
XGBoost	0.867 (±0.017)	0.179 (±0.082)	0.285 (±0.136)	0.992 (±0.005)	0.309 (±0.116)	0.285 (±0.094)
RNN	0.762 (±0.022)	0.456 (±0.007)	0.453 (±0.015)	0.995 (±0.001)	0.500 (±0.022)	0.475 (±0.005)
GRU	0.805 (±0.017)	0.468 (±0.008)	0.464 (±0.010)	0.994 (±0.000)	0.502 (±0.011)	0.482 (±0.007)
LSTM	0.815 (±0.019)	0.466 (±0.003)	0.462 (±0.015)	0.995 (±0.001)	0.506 (±0.011)	0.482 (±0.005)
GRU-D	0.885 (±0.017)	0.472 (±0.012)	0.466 (±0.012)	0.994 (±0.000)	0.504 (±0.014)	0.484 (±0.011)
GRU-D++	0.888 (±0.016)	0.481 (±0.008)	0.474 (±0.018)	0.995 (±0.001)	0.511 (±0.029)	0.491 (±0.005)
ROX	0.631 (±0.026)	0.018 (±0.001)	0.090 (±0.011)	0.966 (±0.006)	0.031 (±0.004)	0.046 (±0.004)
HACOR	0.768 (±0.025)	0.026 (±0.001)	0.122 (±0.063)	0.974 (±0.021)	0.059 (±0.009)	0.075 (±0.002)

Abbreviations: MIMIC-IV, Medical Information Mart for Intensive Care IV; XGBoost, Extreme Gradient Boosting; RNN, Recurrent Neural Network; GRU, Gated Recurrent Unit; LSTM, Long Short-Term Memory; GRU-D, Gated Recurrent Unit with Decay; GRU-D++, Gated Recurrent Unit with Decay++; ROX, Respiratory Rate-Oxygenation index; HACOR, Heart rate, Acidosis, Consciousness, Oxygenation, and Respiratory rate score; AUROC, Area Under the Receiver Operating Characteristic curve; AUPR, Area Under the Precision-Recall curve; F1, F1-score.

**Table A2:** Comparison of various prediction methods on the MIMIC-IV dataset: 4 h in-advance scenario. Thresholds for the models are 0.064, 0.250, 0.370, 0.226, 0.610, 0.624, 0.600, 0.644, 0.632, 0.113, 7.010 (top to bottom).

	AUROC	AUPR	Sensitivity	Specificity	Precision	F1
LogisticRegression	0.711 (±0.027)	0.080 (±0.007)	0.167 (±0.015)	0.976 (±0.003)	0.137 (±0.008)	0.150 (±0.006)
DecisionTree	0.593 (±0.014)	0.128 (±0.002)	0.410 (±0.007)	0.976 (±0.001)	0.279 (±0.003)	0.332 (±0.002)
RandomForest	0.807 (±0.021)	0.451 (±0.006)	0.479 (±0.010)	0.992 (±0.000)	0.577 (±0.007)	0.523 (±0.006)
XGBoost	0.834 (±0.018)	0.274 (±0.156)	0.411 (±0.086)	0.969 (±0.019)	0.289 (±0.191)	0.323 (±0.140)
RNN	0.729 (±0.023)	0.508 (±0.008)	0.481 (±0.008)	0.992 (±0.000)	0.580 (±0.011)	0.526 (±0.004)
GRU	0.791 (±0.019)	0.513 (±0.004)	0.485 (±0.012)	0.992 (±0.001)	0.579 (±0.016)	0.528 (±0.003)
LSTM	0.793 (±0.020)	0.516 (±0.007)	0.485 (±0.009)	0.992 (±0.001)	0.586 (±0.013)	0.531 (±0.003)
GRU-D	0.857 (±0.017)	0.515 (±0.008)	0.470 (±0.017)	0.993 (±0.001)	0.607 (±0.019)	0.529 (±0.007)
GRU-D++	0.859 (±0.016)	0.519 (±0.010)	0.475 (±0.005)	0.993 (±0.000)	0.609 (±0.017)	0.534 (±0.007)
ROX	0.642 (±0.024)	0.036 (±0.001)	0.452 (±0.326)	0.742 (±0.201)	0.044 (±0.009)	0.070 (±0.003)
HACOR	0.736 (±0.024)	0.036 (±0.001)	0.188 (±0.014)	0.930 (±0.008)	0.057 (±0.002)	0.087 (±0.002)

Abbreviations: MIMIC-IV, Medical Information Mart for Intensive Care IV; XGBoost, Extreme Gradient Boosting; RNN, Recurrent Neural Network; GRU, Gated Recurrent Unit; LSTM, Long Short-Term Memory; GRU-D, Gated Recurrent Unit with Decay; GRU-D++, Gated Recurrent Unit with Decay++; ROX, Respiratory Rate-Oxygenation index; HACOR, Heart rate, Acidosis, Consciousness, Oxygenation, and Respiratory rate score; AUROC, Area Under the Receiver Operating Characteristic curve; AUPR, Area Under the Precision-Recall curve; F1, F1-score.

**Table A3:** Comparison of various prediction methods on the MIMIC-IV dataset: 8 h in-advance scenario. Thresholds for the models are 0.066, 0.354, 0.388, 0.190, 0.660, 0.670, 0.666, 0.672, 0.664, 0.145, 5.810 (top to bottom).

	AUROC	AUPR	Sensitivity	Specificity	Precision	F1
LogisticRegression	0.689 (±0.023)	0.091 (±0.006)	0.202 (±0.021)	0.954 (±0.011)	0.130 (±0.013)	0.157 (±0.004)
DecisionTree	0.569 (±0.012)	0.134 (±0.004)	0.395 (±0.009)	0.967 (±0.001)	0.288 (±0.008)	0.333 (±0.006)
RandomForest	0.764 (±0.021)	0.434 (±0.010)	0.427 (±0.010)	0.992 (±0.000)	0.636 (±0.010)	0.511 (±0.009)
XGBoost	0.802 (±0.021)	0.249 (±0.150)	0.381 (±0.112)	0.956 (±0.026)	0.298 (±0.228)	0.311 (±0.150)
RNN	0.784 (±0.022)	0.512 (±0.011)	0.486 (±0.013)	0.987 (±0.001)	0.557 (±0.006)	0.519 (±0.007)
GRU	0.812 (±0.019)	0.519 (±0.006)	0.493 (±0.008)	0.987 (±0.001)	0.557 (±0.009)	0.523 (±0.005)
LSTM	0.808 (±0.020)	0.518 (±0.008)	0.487 (±0.011)	0.987 (±0.001)	0.559 (±0.006)	0.520 (±0.005)
GRU-D	0.833 (±0.019)	0.519 (±0.006)	0.487 (±0.010)	0.987 (±0.001)	0.564 (±0.008)	0.522 (±0.006)
GRU-D++	0.849 (±0.018)	0.521 (±0.010)	0.493 (±0.013)	0.987 (±0.001)	0.558 (±0.012)	0.523 (±0.007)
ROX	0.644 (±0.023)	0.055 (±0.001)	0.714 (±0.012)	0.584 (±0.025)	0.055 (±0.003)	0.102 (±0.005)
HACOR	0.704 (±0.023)	0.049 (±0.001)	0.192 (±0.018)	0.919 (±0.010)	0.074 (±0.002)	0.107 (±0.003)

Abbreviations: MIMIC-IV, Medical Information Mart for Intensive Care IV; XGBoost, Extreme Gradient Boosting; RNN, Recurrent Neural Network; GRU, Gated Recurrent Unit; LSTM, Long Short-Term Memory; GRU-D, Gated Recurrent Unit with Decay; GRU-D++, Gated Recurrent Unit with Decay++; ROX, Respiratory Rate-Oxygenation index; HACOR, Heart rate, Acidosis, Consciousness, Oxygenation, and Respiratory rate score; AUROC, Area Under the Receiver Operating Characteristic curve; AUPR, Area Under the Precision-Recall curve; F1, F1-score.

**Table A4:** Comparison of various prediction methods on the KNUH dataset: 2 h in-advance scenario. Thresholds for the models are 0.070, 0.522, 0.234, 0.534, 0.460, 0.428, 0.398, 0.404, 0.434, 0.053, 13.010 (top to bottom).

	AUROC	AUPR	Sensitivity	Specificity	Precision	F1
LogisticRegression	0.745 (±0.022)	0.024 (±0.000)	0.145 (±0.018)	0.992 (±0.001)	0.050 (±0.002)	0.075 (±0.001)
DecisionTree	0.579 (±0.014)	0.004 (±0.000)	0.143 (±0.010)	0.930 (±0.008)	0.006 (±0.001)	0.012 (±0.001)
RandomForest	0.762 (±0.021)	0.007 (±0.000)	0.206 (±0.078)	0.924 (±0.035)	0.009 (±0.001)	0.016 (±0.001)
XGBoost	0.797 (±0.018)	0.009 (±0.005)	0.072 (±0.029)	0.992 (±0.004)	0.034 (±0.016)	0.044 (±0.017)
RNN	0.870 (±0.016)	0.050 (±0.011)	0.176 (±0.025)	0.995 (±0.003)	0.105 (±0.031)	0.127 (±0.020)
GRU	0.897 (±0.013)	0.080 (±0.015)	0.181 (±0.018)	0.997 (±0.001)	0.153 (±0.030)	0.164 (±0.019)
LSTM	0.893 (±0.013)	0.068 (±0.012)	0.191 (±0.026)	0.996 (±0.002)	0.124 (±0.024)	0.148 (±0.013)
GRU-D	0.856 (±0.018)	0.064 (±0.010)	0.177 (±0.022)	0.996 (±0.000)	0.124 (±0.010)	0.146 (±0.014)
GRU-D++	0.913 (±0.013)	0.063 (±0.008)	0.162 (±0.018)	0.997 (±0.001)	0.137 (±0.021)	0.147 (±0.012)
ROX	0.678 (±0.021)	0.010 (±0.000)	0.129 (±0.000)	0.984 (±0.000)	0.023 (±0.000)	0.040 (±0.000)
HACOR	0.768 (±0.019)	0.013 (±0.000)	0.129 (±0.000)	0.986 (±0.000)	0.028 (±0.000)	0.046 (±0.000)

Abbreviations: KNUH, Kangwon National University Hospital; XGBoost, Extreme Gradient Boosting; RNN, Recurrent Neural Network; GRU, Gated Recurrent Unit; LSTM, Long Short-Term Memory; GRU-D, Gated Recurrent Unit with Decay; GRU-D++, Gated Recurrent Unit with Decay++; ROX, Respiratory Rate-Oxygenation index; HACOR, Heart rate, Acidosis, Consciousness, Oxygenation, and Respiratory rate score; AUROC, Area Under the Receiver Operating Characteristic curve; AUPR, Area Under the Precision-Recall curve; F1, F1-score.

**Table A5:** Comparison of various prediction methods on the KNUH dataset: 4 h in-advance scenario. Thresholds for the models are 0.100, 0.666, 0.376, 0.326, 0.534, 0.582, 0.456, 0.554, 0.584, 0.063, 12.010 (top to bottom).

	AUROC	AUPR	Sensitivity	Specificity	Precision	F1
LogisticRegression	0.706 (±0.026)	0.029 (±0.000)	0.123 (±0.002)	0.990 (±0.000)	0.066 (±0.001)	0.086 (±0.001)
DecisionTree	0.564 (±0.013)	0.007 (±0.000)	0.184 (±0.009)	0.905 (±0.004)	0.011 (±0.001)	0.021 (±0.001)
RandomForest	0.755 (±0.021)	0.013 (±0.001)	0.127 (±0.057)	0.956 (±0.027)	0.018 (±0.003)	0.030 (±0.003)
XGBoost	0.756 (±0.021)	0.014 (±0.003)	0.091 (±0.031)	0.982 (±0.009)	0.033 (±0.015)	0.045 (±0.012)
RNN	0.807 (±0.020)	0.065 (±0.007)	0.191 (±0.038)	0.991 (±0.002)	0.110 (±0.014)	0.137 (±0.011)
GRU	0.854 (±0.017)	0.086 (±0.005)	0.154 (±0.047)	0.995 (±0.004)	0.160 (±0.032)	0.149 (±0.010)
LSTM	0.862 (±0.016)	0.075 (±0.010)	0.218 (±0.067)	0.988 (±0.007)	0.114 (±0.038)	0.138 (±0.015)
GRU-D	0.863 (±0.017)	0.087 (±0.007)	0.182 (±0.087)	0.992 (±0.007)	0.164 (±0.068)	0.146 (±0.013)
GRU-D++	0.881 (±0.014)	0.089 (±0.007)	0.149 (±0.020)	0.996 (±0.001)	0.168 (±0.019)	0.157 (±0.013)
ROX	0.686 (±0.024)	0.017 (±0.000)	0.153 (±0.000)	0.976 (±0.000)	0.035 (±0.000)	0.057 (±0.000)
HACOR	0.716 (±0.023)	0.016 (±0.000)	0.118 (±0.000)	0.981 (±0.000)	0.034 (±0.000)	0.053 (±0.000)

Abbreviations: KNUH, Kangwon National University Hospital; XGBoost, Extreme Gradient Boosting; RNN, Recurrent Neural Network; GRU, Gated Recurrent Unit; LSTM, Long Short-Term Memory; GRU-D, Gated Recurrent Unit with Decay; GRU-D++, Gated Recurrent Unit with Decay++; ROX, Respiratory Rate-Oxygenation index; HACOR, Heart rate, Acidosis, Consciousness, Oxygenation, and Respiratory rate score; AUROC, Area Under the Receiver Operating Characteristic curve; AUPR, Area Under the Precision-Recall curve; F1, F1-score.

**Table A6:** Comparison of various prediction methods on the KNUH dataset: 8 h in-advance scenario. Thresholds for the models are 0.108, 0.582, 0.350, 0.122, 0.384, 0.494, 0.450, 0.446, 0.434, 0.073, 10.010 (top to bottom).

	AUROC	AUPR	Sensitivity	Specificity	Precision	F1
LogisticRegression	0.690 (±0.033)	0.036 (±0.000)	0.136 (±0.008)	0.984 (±0.002)	0.073 (±0.002)	0.095 (±0.000)
DecisionTree	0.547 (±0.011)	0.011 (±0.000)	0.210 (±0.005)	0.875 (±0.008)	0.016 (±0.001)	0.029 (±0.002)
RandomForest	0.753 (±0.024)	0.018 (±0.001)	0.218 (±0.135)	0.908 (±0.069)	0.035 (±0.029)	0.041 (±0.003)
XGBoost	0.753 (±0.026)	0.028 (±0.009)	0.264 (±0.175)	0.908 (±0.130)	0.048 (±0.022)	0.073 (±0.025)
RNN	0.780 (±0.026)	0.068 (±0.014)	0.310 (±0.056)	0.967 (±0.012)	0.086 (±0.015)	0.132 (±0.012)
GRU	0.798 (±0.026)	0.092 (±0.007)	0.236 (±0.065)	0.982 (±0.008)	0.121 (±0.036)	0.151 (±0.006)
LSTM	0.805 (±0.024)	0.092 (±0.004)	0.257 (±0.042)	0.981 (±0.005)	0.114 (±0.012)	0.156 (±0.006)
GRU-D	0.774 (±0.028)	0.099 (±0.008)	0.262 (±0.058)	0.981 (±0.007)	0.118 (±0.015)	0.160 (±0.007)
GRU-D++	0.815 (±0.024)	0.097 (±0.004)	0.277 (±0.057)	0.979 (±0.007)	0.114 (±0.015)	0.159 (±0.005)
ROX	0.705 (±0.029)	0.026 (±0.000)	0.188 (±0.000)	0.967 (±0.000)	0.051 (±0.000)	0.080 (±0.000)
HACOR	0.693 (±0.029)	0.020 (±0.000)	0.147 (±0.000)	0.965 (±0.000)	0.038 (±0.000)	0.061 (±0.000)

Abbreviations: KNUH, Kangwon National University Hospital; XGBoost, Extreme Gradient Boosting; RNN, Recurrent Neural Network; GRU, Gated Recurrent Unit; LSTM, Long Short-Term Memory; GRU-D, Gated Recurrent Unit with Decay; GRU-D++, Gated Recurrent Unit with Decay++; ROX, Respiratory Rate-Oxygenation index; HACOR, Heart rate, Acidosis, Consciousness, Oxygenation, and Respiratory rate score; AUROC, Area Under the Receiver Operating Characteristic curve; AUPR, Area Under the Precision-Recall curve; F1, F1-score.

**Table A7:** Exact *p*-values of baseline characteristics.

	MIMIC-IV	KNUH
Age	2.01644 × 10^−5^	0.033157189
Sex	3.1132 × 10^−228^	7.92096 × 10^−12^
Body Temperature	8.7673 × 10^−128^	1.03122 × 10^−28^
SBP	1.00993 × 10^−51^	0.96353178
DBP	3.12711 × 10^−77^	0.000493843
Heart Rate	5.0527 × 10^−304^	7.2444 × 10^−162^
Respiratory Rate	5.2665 × 10^−162^	5.22767 × 10^−66^
PaCO_2_	4.19061 × 10^−63^	1.54311 × 10^−8^
Lactate	0	3.68074 × 10^−49^
pH	6.2709 × 10^−214^	4.29779 × 10^−55^
Bicarbonate	2.07323 × 10^−61^	2.51427 × 10^−12^
P/F Ratio	0	1.2025 × 10^−166^
GCS (motor)	0	2.4113 × 10^−257^
GCS (verbal)	0	1.84847 × 10^−27^
GCS (eye)	0	1.807 × 10^−277^

Abbreviations: MIMIC-IV, Medical Information Mart for Intensive Care IV; KNUH, Kangwon National University Hospital; SBP, Systolic Blood Pressure; DBP, Diastolic Blood Pressure; P/F Ratio, PaO_2_/FiO_2_ ratio; GCS, Glasgow Coma Scale.

## References

[1] Schwartz D.E., Matthay M.A., Cohen N.H. (1995). Death and other complications of emergency airway management in critically ill adults. A prospective investigation of 297 tracheal intubations. Anesthesiology.

[2] Le Gall J.R., Loirat P., Alperovitch A., Glaser P., Granthil C., Mathieu D., Mercier P., Thomas R., Villers D. (1984). A simplified acute physiology score for ICU patients. Crit. Care Med..

[3] Van der Merwe E., Kidd M., Meltzer S., Bolliger C.T., Irusen E.M. (2005). Validating the use of the Apache II score in a tertiary South African ICU. S. Afr. J. Crit. Care.

[4] Knaus W.A., Wagner D.P., Draper E.A., Zimmerman J.E., Bergner M., Bastos P.G., Sirio C.A., Murphy D.J., Lotring T., Damiano A. (1991). The Apache III prognostic system: Risk prediction of hospital mortality for critically III hospitalized adults. Chest.

[5] Zimmerman J.E., Kramer A.A., McNair D.S., Malila F.M. (2006). Acute Physiology and Chronic Health Evaluation (APACHE) IV: Hospital mortality assessment for today’s critically ill patients. Crit. Care Med..

[6] Singer M., Deutschman C.S., Seymour C.W., Shankar-Hari M., Annane D., Bauer M., Bellomo R., Bernard G.R., Chiche J.D., Coopersmith C.M. (2016). The third international consensus definitions for sepsis and septic shock (Sepsis-3). JAMA.

[7] Moreno R., Vincent J.L., Matos R., Mendonça A., Cantraine F., Thijs L., Takala J., Sprung C., Antonelli M., Bruining H. (1999). The use of maximum SOFA score to quantify organ dysfunction/failure in intensive care. Results of a prospective, multicentre study. Working Group on Sepsis related Problems of the ESICM. Intensive Care Med..

[8] Teasdale G., Jennett B. (1974). Assessment of coma and impaired consciousness: A practical scale. Lancet.

[9] Teasdale G., Maas A., Lecky F., Manley G., Stocchetti N., Murray G. (2014). The Glasgow Coma Scale at 40 years: Standing the test of time. Lancet Neurol..

[10] Vincent J.-L., Moreno R. (2010). Clinical review: Scoring systems in the critically ill. Crit. Care.

[11] Duan J., Han X., Bai L., Zhou L., Huang S. (2017). Assessment of heart rate, acidosis, consciousness, oxygenation, and respiratory rate to predict noninvasive ventilation failure in hypoxemic patients. Intensive Care Med..

[12] Roca O., Messika J., Caralt B., García-de-Acilu M., Sztrymf B., Ricard J.D., Masclans J.R. (2016). Predicting success of high-flow nasal cannula in pneumonia patients with hypoxemic respiratory failure: The utility of the ROX index. J. Crit. Care.

[13] Liu J., Chen X.X., Fang L., Li J.X., Yang T., Zhan Q., Tong K., Fang Z. (2018). Mortality prediction based on imbalanced high-dimensional ICU big data. Comput. Ind..

[14] Shillan D., Sterne J.A.C., Champneys A., Gibbison B. (2019). Use of machine learning to analyse routinely collected intensive care unit data: A systematic review. Crit. Care.

[15] Siu B.M.K., Kwak G.H., Ling L., Hui P. (2020). Predicting the need for intubation in the first 24 h after critical care admission using machine learning approaches. Sci. Rep..

[16] Zhu Y., Zhang J., Wang G., Yao R., Ren C., Chen G., Jin X., Guo J., Liu S., Zheng H. (2021). Machine learning prediction models for mechanically ventilated patients: Analyses of the MIMIC-III database. Front. Med..

[17] Ghauri S.K., Javaeed A., Mustafa K.J., Khan A.S. (2019). Predictors of prolonged mechanical ventilation in patients admitted to intensive care units: A systematic review. Int. J. Health Sci..

[18] Kim Y., Kim H., Choi J., Cho K., Yoo D., Lee Y., Park S.J., Jeong M.H., Jeong S.H., Park K.H. (2023). Early prediction of need for invasive mechanical ventilation in the neonatal intensive care unit using artificial intelligence and electronic health records: A clinical study. BMC Pediatr..

[19] Kim M., Kim T.H., Kim D., Lee D., Kim D., Heo J., Kang S., Ha T., Kim J., Moon D.H. (2023). In-advance prediction of pressure ulcers via deep-learning-based robust missing value imputation on real-time intensive care variables. J. Clin. Med..

[20] Johnson A.E.W., Bulgarelli L., Shen L., Gayles A., Shammout A., Horng S., Pollard T.J., Hao S., Moody B., Gow B. (2023). MIMIC-IV, a freely accessible electronic health record dataset. Sci. Data.

[21] Che Z., Purushotham S., Cho K., Sontag D., Liu Y. (2018). Recurrent neural networks for multivariate time series with missing values. Sci. Rep..

[22] Lundberg S.M., Lee S.-I. (2017). A unified approach to interpreting model predictions. Adv. Neural Inf. Process. Syst..

[23] Duan J., Chen L., Liu X., Bozbay S., Liu Y., Wang K., Esquinas A.M., Shu W., Yang F., He D. (2022). An updated HACOR score for predicting the failure of noninvasive ventilation: A multicenter prospective observational study. Crit. Care.

[24] Roca O., Caralt B., Messika J., Samper M., Sztrymf B., Hernández G., García-de-Acilu M., Frat J.P., Masclans J.R., Ricard J.D. (2019). An index combining respiratory rate and oxygenation to predict outcome of nasal high-flow therapy. Am. J. Respir. Crit. Care Med..

[25] El-Rashidy N., Tarek Z., Elshewey A.M., Shams M.Y. (2025). Multitask multilayer-prediction model for predicting mechanical ventilation and the associated mortality rate. Neural Comput. Appl..

[26] Duan J., Wang S., Liu P., Han X., Tian Y., Gao F., Zhou J., Mou J., Qin Q., Yu J. (2019). Early prediction of noninvasive ventilation failure in COPD patients: Derivation, internal validation, and external validation of a simple risk score. Ann. Intensive Care.

[27] Stoian M., Azamfirei L., Stîngaciu A.C., Negulici L.-M., Văsieșiu A.M., Manea A., Stoian A. (2025). Early Diagnostic Markers and Risk Stratification in Sepsis: Prognostic Value of Neutrophil-to-Lymphocyte Ratio, Platelets, and the Carmeli Score. Biomedicines.

